# Cemented versus uncemented total knee arthroplasty in younger patients: A protocol of retrospective cohort trial

**DOI:** 10.1097/MD.0000000000020087

**Published:** 2020-05-01

**Authors:** Yuning Guo, Shufang Ma, Junbo Wang, Qin Zhang, Shaowei Wang, Zhipo Du

**Affiliations:** a Department of orthopaedics, Yuncheng central hospital, Eighth Clinical Medical College of ShanXi Medical University. ShanXi; b Department of rheumatology and immunology, The 4th Central Hospital of Baoding City, Hebei; c Department of Radiology, Yuncheng central hospital, Eighth Clinical Medical College of ShanXi Medical University; d The Second Hospital of Shanxi Medical University, ShanXi; e Department of orthopaedics, The 4th Central Hospital of Baoding City, Hebei, China.

**Keywords:** cemented, cementless, complication, protocol, revision, total knee arthroplasty

## Abstract

**Background:**

Recently, controversy still exists regarding the clinical effects of cemented or cementless technique in young patients in total knee arthroplasty (TKA). In this context, the present study aimed to determine the functional outcomes and clinical reliability of cementless components versus those of conventional cemented components for young patients in primary TKA.

**Methods:**

A retrospective review of primary TKAs performed with cementless or cemented fixation between May 2010 and February 2019 was conducted with Institutional Review Board approval. All cases were performed by a single surgeon. Institutional review board approval was obtained prior to conducting chart review and analysis. The primary outcome compared between the 2 fixation groups was the rate of postoperative complications and revision related to TKA, occurring at any point in follow-up. Secondary outcome measures included surgical time, Oxford Knee Score, range of motion, and radiographic outcomes such as progressive radiolucent lines, osteolysis, or component migration.

**Results:**

We were able to directly compare the outcomes of cemented versus cementless techniques and might reveal a better technique in TKA.

**Trial registration:**

This study protocol was registered in Research Registry (researchregistry5459).

## 1 Introduction

Arthritic disease in active, younger patients (≤ 55 years) is not uncommon and its prevalence is expected to increase.^[1]^ A number of surgical options for the arthritic knee exist for these patients. Arthroscopic débridement, realignment osteotomy, and arthrodesis are all surgical options that have been proposed for this difficult problem. Many of these treatments, however, provide only short-term relief of symptoms or compromise function.^[2,3]^ More recently, there has been a trend of offering knee arthroplasty as an option to provide pain relief and improve function in the active, younger patient with knee osteoarthritis.^[4]^ Total knee arthroplasty (TKA) has proven to be an effective treatment in young patients, although they are more active and demanding, and thus, more mechanical complications and potential revisions could be expected over time.^[5,6]^ The commonest mechanisms of failure are aseptic tibial loosening, and polyethylene wear and osteolysis.

The optimal mode of fixation in TKA has been an area of debate for decades. Cementless prostheses remain an intriguing option because of the potential for biologic fixation and improved survivorship.^[7]^ However, numerous prior reports of cementless TKA designs have raised concerns regarding failure of fixation, early failure, and poor clinical outcomes.^[7–10]^ Furthermore, the immediate fixation and excellent survivorship of cemented TKA make transitioning from this technique difficult for the majorityof surgeons. Lastly, cementless prostheses are typically more expensive than their cemented counterparts, which can impact a surgeon's choice of the mode of fixation.

Recently, controversy still exists regarding the clinical effects of cemented or cementless technique in young patients in TKA.^[6,11]^ Due to a lack of direct comparison between the clinical outcomes of these 2 techniques in current literature, uncertainty remains regarding the superiority of either method. The purpose of this study was to evaluate:

(1) implant survivorship,
(2) patient functional outcomes, and
(3) complications in younger patients who underwent TKA with either cemented or cementless technique by the same experienced surgeon.

The hypothesis was that the cementless technique would achieve better functional scores and fewer complications as compared to the cemented technique in conventional TKA.

## 2 Materials and methods

### 2.1 Study design and population

A retrospective review of primary TKAs performed with cementless or cemented fixation between May 2010 and February 2019 was conducted with Institutional Review Board approval. All cases were performed by a single surgeon. Institutional review board approval was obtained prior to conducting chart review and analysis (SHX001020). This study was also registered in the Research Registry (researchregistry5459). Inclusion criteria were the following: patients with aged 55 or younger, primary TKA, femorotibial and patellofemoral osteoarthritis, and available clinical and radiographic follow-up for at least 12 months. Exclusion criteria were the following: revision TKA, unicompartmental knee arthroplasty, prior history of open knee surgery or ligament reconstruction on the operative knee, primary TKAs indicated for inflammatory arthritis, and patients with metabolic bone disease.

### 2.2 Operative techniques

Surgeries were performed using a median parapatellar approach. A tourniquet was used from the surgical incision until the postoperative compression dressing was applied. Conventional extramedullary alignment guides were used to cut the tibia in all cases. Femoral cuts were made with an articulating surface mounted navigation system, which avoided pin placement outside of the arthrotomy to enact the distal femoral cut without violation of the intramedullary canal. After the distal femoral cut was made, the remaining operation proceeded in standard fashion with conventional instrumentation. After component cementation or impaction of cementless components, a periarticular injection of epinephrine, ropivacaine, and ketorolac was injected into the retinaculum and synovium except the posterior capsule.

#### 2.2.1 Cemented group

The cemented group consisted of a posterior-stabilized or cruciate-retaining Stryker Triathlon total knee with a cemented all polyethylene patella component. The cementless, screwless, tibial baseplate was developed from pure titanium powder using additive manufacturing technology which can optimize porosity for ingrowth and provide solid material for strength in addition to manufacturing complex geometries. Mechanical testing of this cementless tibial baseplate demonstrated excellent resistance to lift off.

#### 2.2.2 Cementless group

All primary cementless knee arthroplasties were performed using a parapatellar or subvastus approach and a posterior-stabilized Stryker Triathlon Tritanium tibial baseplate along with a cementless periapatite-coated femoral component, a cementless patella component, and cross-linked polyethylene liner.

### 2.3 Postoperative care

A medium hemovac drain was placed and removed on postoperative day 1. Aspirin, or low dose warfarin was used for postoperative thromboprophylaxis, with patients being given 5 mg warfarin orally on the night of surgery with subsequent daily adjustments as needed to maintain an international normalized ratio between 1.8 and 2.2. All patients received the same standardized postoperative multimodal pain protocol, with four doses of 1 g of acetaminophen, two doses of celecoxib 200 mg, and morphine (first 48 hours) or tramadol (after 48 hours) for pain exacerbations. All patients underwent the same postoperative rehabilitation program, with partial weight bearing with the use of crutches for the first postoperative day and active range of movement exercises. All patients received the same preoperative antibiotic protocol.

### 2.4 Clinical outcome measures

The primary outcome compared between the 2 fixation groups was the rate of postoperative complications and revision related to TKA, occurring at any point in follow-up. Complications included major postoperative events such as revision surgery for any indication, aseptic loosening of any component, periprosthetic fractures, deep vein thrombosis, prosthetic joint infection, manipulation under anesthesia, surgical lysis of adhesions for knee stiffness, and local tissue irritation associated with TKA such as pes anserine bursitis. Secondary outcome measures included surgical time, Oxford Knee Score (OKS), range of motion, and radiographic outcomes such as progressive radiolucent lines, osteolysis, or component migration. The OKS, range of motion, postoperative complications, and revision were obtained both before and after surgery at a minimum of 3 years postoperatively. Subjects were asked to complete an OKS questionnaire and rate their satisfaction with their TKA. The OKS consists of 12 questions assessed on a Likert scale with values from 0 to 4, a summative score is then calculated where 48 is the best possible score (least symptomatic) and 0 is the worst possible score (most symptomatic). For patients who were not seen recently, the scores were obtained via telephone. Postoperative complications and revision procedures were documented during routine collection of follow-up data. All data were independently verified by a detailed review of hospital operative reports, anesthesia records, and clinical records. Data were abstracted by 1 of 2 research personnel blinded to patient group and study aim.

### 2.5 Statistical analysis

Statistical analysis was performed using SPSS version. Continuous outcome variables and their difference were tested with parametrical statistical techniques, such as *t* tests, unless the normality test showed a nonparametric distribution of the data, in which case a Mann-Whitney test was used. Survival was analysed using the Kaplan-Meier survival analysis. Significance for survival was calculated using the generalized Wilcoxon test. Categorical outcome variables were analysed with the Chi-squared test. *P* values < .05 were considered statistically significant. The power calculation showed that if the experimental and control partial radiolucency rates are in accordance with the literature, we would need to study at least 38 experimental subjects and 38 control subjects to be able to reject the null hypothesis that the radiolucency rate of the experimental and control groups is equal with probability (power) 0.8. The Type I error probability associated with the test of this null hypothesis is 0.05.

## 3 Result

The results will be shown in Tables 1 and 2.

**Table 1:**
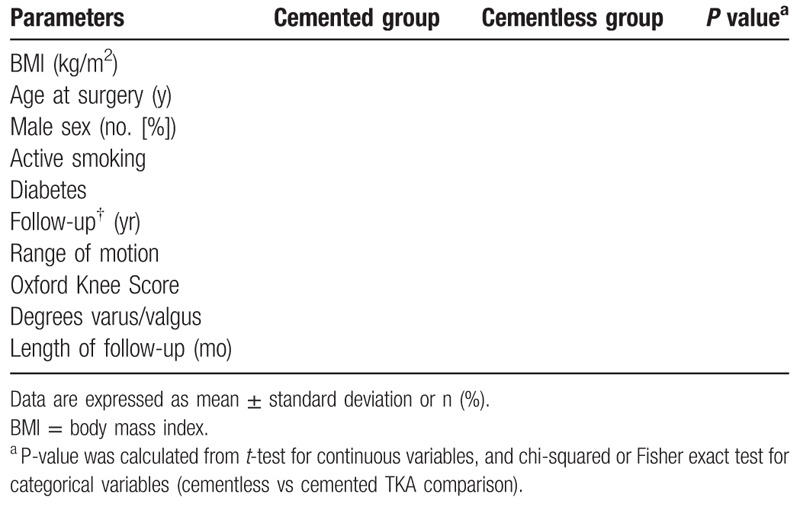
Pre-operative data.

**Table 2:**
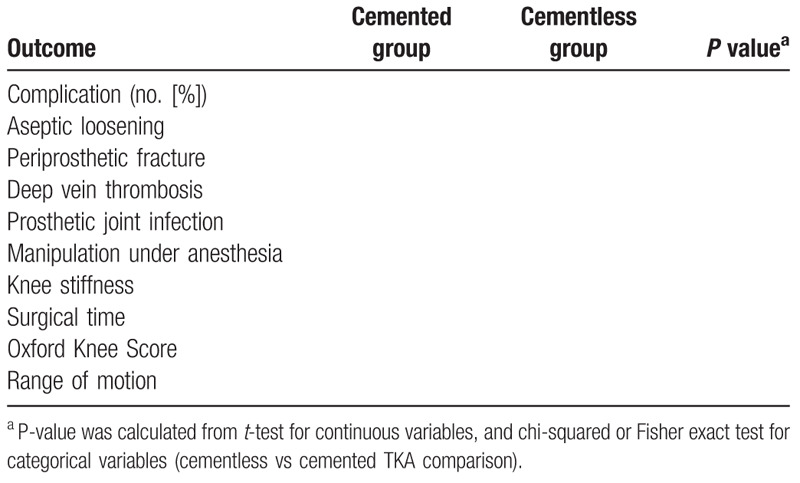
The postoperative outcomes in the 2 groups.

## 4 Discussion

Cemented TKAs show low rates of aseptic loosening in long-term follow-up, with good clinical outcomes. However, observed signs of osteolysis at the cement-bone interface have raised questions about the long-term durability of cemented TKAs in younger patients.^[12,13]^ Cementless TKAs have been developed in an attempt to improve the longevity of implants, particularly in younger patients. It is thought that a more physiological bond between bone and implant would result in improved survival from aseptic loosening. Nevertheless, the signs of osteolysis also have been observed with cementless TKAs.^[14–16]^

To date, there are few cohort trials in which cemented and cementless fixation in primary TKAs are compared in young patient groups. To our knowledge, it is currently controversial whether cementless fixation or conventional cemented fixation is superior in terms of postoperative functional recovery, implant survival, total complication, and radiological performance for primary TKA in young patients. In this context, the present study aimed to determine the functional outcomes and clinical reliability of cementless components versus those of conventional cemented components for young patients in primary TKA.

Limitations of this study included single surgeon practice, single implant manufacturer, and single implant model utilized, lack of patient randomization, and no advanced imaging (computed tomography scan) for accurate preoperative and postoperative measurements. In addition, the limitations of our study also included those inherent in any retrospective cohort study, including the possibility of selection or observational bias. Despite the above mentioned limitations of this study, we were able to directly compare the outcomes of cemented versus cementless techniques and might reveal a better technique in TKA.

## Author contributions

**Conceptualization:** Yuning Guo, Shufang Ma.

**Data curation:** Yuning Guo, Shufang Ma.

**Formal analysis:** Yuning Guo, Qin Zhang, Junbo Wang.

**Funding acquisition:** Zhipo Du, Shaowei Wang, Qin Zhang.

**Investigation:** Yuning Guo, Shufang Ma, Qin Zhang, Junbo Wang.

**Methodology:** Yuning Guo, Zhipo Du, Shaowei Wang.

**Resources:** Zhipo Du.

**Software:** Zhipo Du.

**Supervision:** Zhipo Du, Shaowei Wang.

**Validation:** Qin Zhang, Junbo Wang.

**Visualization:** Qin Zhang, Junbo Wang.

**Writing – original draft:** Yuning Guo, Qin Zhang, Junbo Wang.

**Writing – review and editing:** Yuning Guo, Qin Zhang, Junbo Wang, Zhipo Du.

Zhipo Du orcid: 0000-0003-0841-3056.
